# Addressing production gaps for vaccines in African countries

**DOI:** 10.2471/BLT.21.287381

**Published:** 2021-11-17

**Authors:** Anna Mia Ekström, Göran Tomson, Rhoda K Wanyenze, Zulfiqar A Bhutta, Catherine Kyobutungi, Agnes Binagwaho, Ole Petter Ottersen

**Affiliations:** a Department of Global Public Health, Karolinska Institutet, 171 77 Stockholm, Sweden.; b Swedish Institute for Global Health Transformation, SIGHT, Royal Swedish Academy of Sciences, Stockholm, Sweden.; c College of Health Sciences, School of Public Health, Makerere University, Uganda.; d Institute for Global Health & Development, The Aga Khan University, Karachi, Pakistan.; e African Population and Health Research Center, Nairobi, Kenya.; f University of Global Health Equity, Kigali, Rwanda.; g President’s Office, Karolinska Institutet, Stockholm, Sweden.

Global health initiatives rely on international solidarity. However, the extreme inequity in access to vaccines for coronavirus disease 2019 (COVID-19) across countries demonstrates that we cannot depend on national politicians and industry alone to make strategic choices for our global common good. High-income countries have been accused of undermining the coordinated purchase and equitable distribution of COVID-19 vaccines through non-transparent pharmaceutical deals, production delays and vaccine export restrictions.^1^ As of 1 November 2021 fewer than 35 million of over 7 billion COVID-19 vaccine doses have been administered in low-income countries.^2^ Nevertheless, high-income countries have disregarded the World Health Organization’s (WHO) plea to pause booster doses of vaccines to give low- and middle-income countries a chance to vaccinate their most vulnerable populations. Despite these challenges, vaccine allocation to low- and middle-income countries through COVAX, the COVID-19 Vaccines Global Access initiative, is finally accelerating. Even so, only five African countries, fewer than 10% of its 54 nations, are expected to reach the year-end target of fully vaccinating 40% of their population,^3^ a goal that would require 800 million more doses of vaccine. Meanwhile, high-income countries will have over 1 billion surplus doses of vaccine.^4^

Africa currently imports 99% of its vaccines^5^ and by November 2021 fewer than 123 million (10%) of 1373 million Africans have been fully or partly immunized against COVID-19.^2^ This deficiency in local vaccine production in Africa and other low- and middle-income countries is a result of chronic underinvestment in research and development, poor knowledge transfer, imbalanced scientific exchange and emigration of highly trained professionals. The annual loss to the African health sector of investment in trained doctors is around 2 billion United States dollars (US$).^6^ This current dependency on a limited number of vaccine manufacturers, with just four companies controlling about 90% of the global vaccine market,^1^ has contributed to an unsustainable pandemic response, a problem that was seen during the 2009 H1N1 influenza pandemic and the 2014 Ebola virus disease outbreak. As the Director-General of the World Trade Organization (WTO) declared in 2021, *“*We have now seen that over-centralization of vaccine production capacity is incompatible with equitable access in a crisis situation.”^7^

Initiatives to reverse this trend are now in progress, accelerated by the COVID-19 pandemic. These initiatives include a new international treaty on pandemics proposed at the Seventy-sixth session of the United Nations General Assembly, the new WHO Hub for Pandemic and Epidemic Intelligence,^8^ and the Multilateral Leaders Task Force (comprising the World Bank, WHO, WTO and International Monetary Fund). The task force urges high-income countries to swap their near-term delivery schedules of COVID-19 vaccines, release vaccine companies from contracts and fulfil their donation pledges, while appealing to pharmaceutical companies to prioritize their contracts with COVAX and the African Vaccine Acquisition Trust.^9^

Until recently, there were only 10 vaccine manufacturers across five African countries –Egypt, Morocco, Senegal, South Africa and Tunisia – jointly producing a tiny fraction of the continent’s needs. Most of these countries have undertaken so-called fill and finish packaging and labelling with very limited upstream production of antigen formulations, principally due to a lack of local scientific capacity, along with weaknesses in the commodity supply chain. These obstacles need to be removed for independence and sustainability of production in Africa. Other key barriers include regulatory hindrances, powerful trade blocs, scarcity of potential purchasers, competition with subsidized markets, political instability, geographical and logistical challenges, lack of sustainable financing mechanisms and low economic purchasing power.^1^^,^^9^ The COVID-19 pandemic is driving new production initiatives for vaccine hubs in Rwanda, Senegal and South Africa and potentially Algeria and Nigeria.^5^^,^^7^ WHO has supported technology-transfer hubs in South Africa to scale up production of mRNA (messenger ribonucleic acid)-based vaccines and stimulate regional development of other pharmaceutical products. These are interesting examples of collaboration between the Africa Centres For Disease Control and Prevention, African universities and pharmaceutical companies. A similar initiative is the African Union’s new Partnerships for African Vaccine Manufacturing, which aims to bolster vaccine manufacturing, financial partnerships, regulatory systems and technology transfer, with African universities becoming vaccine research and development hubs.^10^ However, the research and development goal should not get overlooked in the rush to set up new manufacturing hubs.

Regional research and development and production of essential drugs, diagnostics and vaccines in Africa is necessary to secure ownership and a sustainable supply of products and to enable a quicker response to future infectious agents. A regional focus could also lead to discoveries in diagnostics and therapeutics for current unmet burdens of disease in low- and middle-income countries, settings of low priority for global pharmaceutical companies.^1^ Researchers even argued that the price of locally produced vaccines could become lower than the price that large companies currently charge governments or global partners.^1^ We argue, however, that the production of essential supplies cannot depend on current resource availability. Even before the drop in childhood immunization coverage during the pandemic, 9.4 million African children annually were not receiving their final dose of the diphtheria, tetanus and pertussis vaccine.^5^ When delivered in full, diphtheria–tetanus–pertussis and other vaccine programmes are currently estimated to save two million to three million children’s lives globally each year, a figure which highlights the gaps in countries’ ability both to deliver and to access vaccines.^5^ Ensuring regional self-sufficiency and manufacturing capabilities in regions with the highest burden of infectious diseases is fundamental for future pandemic preparedness and rapid crisis response.

The emergence of mRNA-based technologies could lower the threshold for scaling up regional manufacturing through reduced upfront costs and more rapid adaptation of vaccines to new variants and pathogens. Currently some mRNA vaccines require ultra-cold chain storage and distribution. Although future technologies may avoid this requirement, investments in ultra-cold chains for COVID-19 vaccines across Africa could serve multiple purposes if upcycled into future research laboratories and facilities for storage of new mRNA vaccines. mRNA technology also offers the potential to develop new or better vaccines, for example for malaria and tuberculosis. We believe that mRNA technology offers opportunities to African countries, and even worldwide, that African researchers should be driving forward.

This approach could become a rewarding investment for companies, governments and funders. Assuming the approval of and steady demand for emerging vaccines, Africa’s publicly funded vaccine market could be worth between US$ 2.4–5.4 billion by 2030.^5^ Africa’s demand for vaccines already makes up about a quarter of global vaccine volume and will increase with the estimated 2.5% annual population growth in the region.^5^ Average gross domestic product in Africa is also expected to grow, creating co-financing opportunities in countries eligible for support from GAVI, the Vaccine Alliance. The World Bank has pledged a 600 million euro investment in Aspen Pharmacare, Africa’s largest pharmaceutical company, with support from the governments of France, Germany and the United States of America. The initiative aims to fund the entire vaccine supply chain for Africa and is part of the World Bank’s enhanced support for global vaccine rollout.^5^ The investment appears to be highly cost-effective, as the global economy is estimated to lose up to US$ 9.2 trillion due to the uncoordinated approach to COVID-19 vaccine access that risks prolonging the pandemic.^5^

Despite promising advances, there is no guarantee that the recent technology-transfer initiatives and research and development investments in Africa will bring about the changes needed. We as researchers must take on a greater responsibility to ensure that new vaccine technologies become a global common good. Achieving this aim requires long-term investments in future local knowledge and expertise. Unfortunately, the COVID-19 pandemic has been a setback to progress in education, especially in Africa. The impact of school closures on Africa’s young generations has created a huge backlog in education, forcing millions of children to drop out of school, causing trillions of dollars in lost lifetime incomes and opportunities. The proportion of children who are out of school has doubled in Africa compared with before the pandemic^11^ and two thirds of low- and middle-income countries included in the World Bank’s Education Finance Watch for 2021 reported education budget cuts since the onset of the pandemic.^12^

Nevertheless, the past years have shown that money can be made available for humanitarian and health crises, and that not investing in education is extremely costly both financially and socially. A lack of funding can therefore no longer be used as an excuse by governments or international organizations not to invest in basic science education or in advanced biotechnology training. Strong support from the scientific community will be necessary to make this happen. We also need to mend a growing distrust of academics and other experts and reinvigorate support for evidence-based decisions in public health, support which has been jeopardized during the COVID-19 pandemic.

Although the world’s response to the most recent pandemic has been a clear example of poor international collaboration, it is also an example of unprecedented scientific success in vaccine innovation. The rapid development and production of COVID-19 vaccines is a great achievement for the pharmaceutical industry. Yet it is a success based on decades of government-funded research conducted primarily at universities. It is unacceptable that the benefits of public investments in scientific progress have been so unevenly distributed globally. As researchers, we have a responsibility to be stewards of this legacy of research, to apply systems thinking across the vaccine value chain and be neutral brokers between multiple actors. In global expert panels, we should argue for sustainable knowledge transfer and long-term investments into local research and development and higher education, and create good examples of true academic partnerships between high-income countries and low- and middle-income countries. For young scholars to benefit from the current promotion of vaccine production in Africa, advancements in legal and regulatory management and health economics are needed, while leveraging African public health professionals’ unique skills in epidemiological surveillance adapted to low-resource contexts.

The global scientific community has never been more active, more united or more motivated for research collaboration and data sharing. Let us as researchers take the lead through a call for action with the goal of converting decades of research into vaccines for all.
